# The relationship between suicidal behavior and hematological inflammatory parameters: a retrospective cohort study

**DOI:** 10.3389/fpsyt.2026.1767546

**Published:** 2026-05-19

**Authors:** Meltem Hazel Şimşek, Ulaş Korkmaz, İskender Aksoy, Sema Çağal

**Affiliations:** 1 Department of Psychiatry, Faculty of Medicine, Giresun University, Giresun, Türkiye; 2 Department of Emergency Medicine, Faculty of Medicine, Giresun University, Giresun, Türkiye; 3 Department of Psychiatry, Bulancak State Hospital, Giresun, Türkiye

**Keywords:** hematologic tests, inflammation, suicidal ideation, suicide, suicide attempted

## Abstract

**Background:**

This study investigated the relationship between hematological inflammatory parameters and suicidal behavior by examining their levels before and at the time of referral, exploring their potential use as early indicators in assessing suicide risk.

**Methods:**

Our sample consisted of 454 individuals who visited the emergency department due to suicidal behavior and 451 control individuals of similar age and gender. Routine hematological parameters(neutrophils, monocytes, lymphocytes, platelets) and indices calculated from these values, such as NLR, MLR, PLR, SII, and SIRI, were evaluated both in the period prior to the year of presentation and at the time of presentation. Clinical characteristics and inflammatory parameters were analyzed for suicide attempts and completed suicides. Logistic regression analyses were performed to assess between-group differences, individual changes over time, and factors predicting suicidal behavior.

**Results:**

In our study, inflammatory parameters were significantly elevated in the suicide behavior group(p<0.001). MLR and SIRI levels showed the strongest association with suicide attempts. Logistic regression analysis revealed that high MLR levels prior to admission increased the risk of suicide attempts by 25 times(OR = 25.2), while high monocyte levels at admission increased this risk by 8 times(OR = 7.7). Suicide-related mortality was associated with male gender, the presence of additional physical illnesses, and the absence of psychiatric evaluation. High MLR(OR = 12.202, p=0.007) and monocyte(OR = 13.113, p< 0.001) levels at the time of admission significantly predicted mortality.

**Conclusion:**

Our findings support the presence of chronic low-grade inflammation in suicidal behavior and indicate that these parameters have the potential to be used as accessible and low-cost biomarkers in the assessment of suicide risk.

## 1 Introduction

Suicide is the intentional act of ending one’s life (1). According to World Health Organization (WHO) data, approximately 800,000 people die by suicide annually (2). Suicide ranks second among causes of death among people aged 10–34 years (3). The number of suicide attempts is substantially higher (4, 5). A significant proportion of people at risk of suicide have not sought help from the health system prior to attempting suicide or dying by suicide, and the risks have not been adequately assessed (6, 7). Therefore, identifying objective and clinically applicable markers for the early detection of suicide risk remains a critical need.

According to the literature, suicide attempts are more prevalent among women, whereas completed suicides are more frequent among men (2, 8). Previous studies have identified several sociodemographic risk factors for suicidal behavior, including gender differences, marital status, and social isolation (9–11). Increasing evidence suggests that biological processes may also contribute to suicide risk.

In clinical practice, suicide risk is assessed based on subjective factors such as previous suicidal ideation or attempts, the presence of psychiatric disorders, and hopelessness. However, these approaches are largely subjective and may be insufficient, particularly in individuals who do not undergo psychiatric evaluation prior to suicidal behavior (6). Therefore, identifying objective and clinically applicable biological markers has become an important research focus. In particular, inflammatory processes are thought to play a critical role in determining suicide risk (12).

Many studies have shown that inflammatory markers are elevated in individuals with suicidal behavior (13–17). Although inflammatory cytokines have been associated with suicidal behavior, their clinical use is limited due to cost and accessibility. Therefore, attention has shifted toward CBC-derived inflammatory markers such as NLR, MLR, PLR, SII, and SIRI. These markers are derived from routine blood count parameters and are widely used in clinical research as indicators of systemic inflammation (18). Additionally, neutrophil, lymphocyte, monocyte, and platelet counts, which are evaluated through routine blood tests, may also indicate inflammation (19, 20). These are commonly used values in the assessment of chronic peripheral inflammation.

Recently defined indices such as SII (neutrophil × platelet/lymphocyte) and SIRI (neutrophil × monocyte/lymphocyte) comprehensively reflect systemic inflammation and provide information about both immune response and inflammatory activity (21–23). Some studies have reported that these indices may be associated with depression, bipolar disorder, and suicide (14, 24–28). However, most previous studies have focused on inflammatory markers measured at the time of suicidal behavior, while data on pre-existing inflammatory status prior to suicidal events remain scarce.In this study, we evaluated inflammatory parameters both within the year preceding suicidal behavior and at the time of presentation. This approach enables a more comprehensive evaluation of both baseline and acute inflammatory processes associated with suicidal behavior (29).

Therefore, this study aimed to investigate whether CBC-derived inflammatory markers measured both prior to and at the time of presentation could serve as early biological markers of suicidal behavior. We hypothesized that parameters such as NLR, MLR, PLR, SII, and SIRI would be significantly associated with suicidal behavior and clinical outcomes. These findings may contribute to the identification of accessible and cost-effective biomarkers for early suicide risk assessment in clinical settings. Integrating such markers into routine clinical evaluation may support clinicians in improving risk stratification and preventive interventions.

## 2 Materials and methods

### 2.1 Data sources

This study is a retrospective cohort study designed and conducted in accordance with the ethical principles of the Helsinki Declaration. Ethical approval for the study was obtained from the Ethics Committee of Giresun Training and Research Hospital (Date: 11.04.2025, Decision No: KAEK/09.04.2025/04). Due to the retrospective nature of the study, the Ethics Committee of Giresun Training and Research Hospital waived the need for obtaining informed consent. The study was conducted by retrospectively reviewing the electronic records of emergency department visits at three different hospitals located in a province of Turkey between January 2020 and December 2024. Data were obtained from the hospital information management system using triage records, ICD codes, patient files, and laboratory results.

All hematological data were obtained using Sysmex XN series automatic hematology analyzers (Sysmex Corporation, Kobe, Japan) in the laboratories of the three hospitals where the study was conducted. These devices are CE-certified and have accuracy and reliability levels suitable for routine clinical use. CBC data prior to admission were determined as those from the previous one-year period. This approach was based on literature findings suggesting that chronic low-grade inflammatory processes may reflect physiological predispositions preceding suicidal behavior (29).

### 2.2 Study population

Individuals who presented to the emergency department due to suicidal behavior and met the inclusion criteria were included in this study. A total of 1,263 individuals were initially screened. After applying the exclusion criteria, 454 individuals were included in the sample group and 451 individuals were included in the control group, resulting in a final sample of 905 individuals. The sample group consisted of individuals who attempted suicide requiring medical intervention (n = 369) and individuals who did not attempt suicide but sought help due to active suicidal ideations (n = 85). The control group consisted of individuals who did not have a psychiatric diagnosis or history of suicide, had at least one complete blood count result in hospital records within the last year, and were similar to the sample group in terms of characteristics such as age and sex. If the same person had multiple applications, only the first application corresponding to the study period was included in the analysis. In our hospital setting, complete blood count (CBC) tests are routinely performed for various administrative and occupational health evaluations, including pre-employment screening, military and law enforcement applications, adoption procedures, and security clearance assessments. These evaluations are typically conducted in individuals without acute illness. Therefore, CBC data for the control group were obtained from individuals undergoing routine health screening rather than for acute or inflammatory conditions.

The criteria for inclusion in the study are as follows: (1) Having presented to the emergency department due to suicidal behavior within the specified timeframe, (2) having a complete CBC result in electronic medical records at the time of presentation, (3) having at least one additional CBC result recorded in the year prior to presentation, (4) being 18 years of age or older at the time of presentation, and (5) having complete medical records related to the study variables available. The individuals included in the control group were selected from among those aged 18 years and older, with no history of psychiatric diagnosis or suicidal behavior, and with at least one CBC result recorded in the past year, in a manner consistent with the sample group in terms of age and gender.

The exclusion criteria for both groups were determined as follows: (1) incomplete or insufficient data in electronic health records, (2) CBC results that were unreliable due to technical problems (e.g., hemolyzed samples, tests containing measurement errors), (3) the presence of active infection, sepsis, acute inflammatory disease, or a history of blood transfusion at the time of blood sample collection, (4) hematological malignancy, active cancer treatment (chemotherapy), or autoimmune diseases that may independently affect hematological parameters, and (5) being under the age of 18. Some of the patients included in the present study may overlap with those reported in a previous study by our group; however, the objectives, study design, and analytical focus of the two studies are distinct (30).

### 2.3 Outcome

The primary objective of this study is to evaluate the relationship between inflammatory parameters in individuals and suicidal behavior (suicidal ideation and attempt). In this context, neutrophil, lymphocyte, monocyte, and platelet counts, along with biomarkers derived from these cells such as NLR, MLR, PLR, SII, and SIRI, were considered as primary variables.

The secondary objective of the study is to examine the relationship between the measured hematological parameters and the clinical outcomes following suicide. These clinical outcomes were determined as hospitalization rate, intensive care requirement, discharge status, and exitus status. The potential of inflammatory parameters at the time of admission and prior to admission to predict these clinical outcomes was evaluated using regression models.

The practical aim of our study is to test the feasibility of using inflammatory markers as a support mechanism in clinical practice for the early detection of suicide risk and the prediction of clinical outcomes.

### 2.4 Independent variables

In our study, independent variables were divided into two main groups: sociodemographic/clinical variables and hematological inflammation markers. Sociodemographic variables included age, gender, marital status, and place of residence, while clinical variables encompassed psychiatric diagnosis history, previous suicide attempts, comorbid physical illnesses, psychiatric medication use, presence of psychiatric consultation at the time of presentation, season, and year of presentation. These variables aim to examine suicidal behavior within its social and clinical context.

The second group of independent variables are hematological inflammation markers derived from CBC. Neutrophil, lymphocyte, monocyte, and platelet counts, as well as composite biomarkers such as NLR, MLR, PLR, SII, and SIRI calculated from these cells, were evaluated. These parameters were analyzed at two time points (the last year prior to presentation and at the time of presentation) to predict biological characteristics and clinical outcomes associated with suicidal behavior. All variables were used in regression models to predict suicide attempts and mortality.

### 2.5 Statistical analysis

Statistical analyses were performed using IBM SPSS Statistics version 27.0. First, the distribution of continuous variables was evaluated in terms of normality. Skewness and kurtosis values within the range of ±1.5 were accepted as indicators of normal distribution (31).

To compare continuous variables among the three groups (suicide attempt, suicidal ideation, and control), one-way analysis of variance (ANOVA) was applied to variables meeting the normality assumption, and *post-hoc* comparisons were performed using Tukey HSD or Games-Howell tests for significant results. For variables that did not follow a normal distribution, the Kruskal–Wallis H test was used, and multiple comparisons were made with Bonferroni correction when necessary. The effect size was calculated as eta-square (η^2^) in ANOVA analyses and epsilon-square (ϵ^2^) in Kruskal–Wallis H analyses.

The chi-square test (Pearson’s Chi-Square Test/Fisher’s exact test) was used to compare categorical variables between groups. Phi (φ) was calculated as the effect size for two-category comparisons, and Cramér’s V was calculated for multi-category comparisons.

In the comparison of measurements belonging to two different time points in the same individuals, depending on the distribution, dependent groups t-test or Wilcoxon signed-rank test were applied. In the dependent groups t-test, Cohen’s d was reported as the effect size, while in the Wilcoxon test, the effect size was reported as the r value.

The effects of inflammatory markers at two different time points (pre-suicide and at the time of presentation) were evaluated using binary logistic regression analysis to predict suicide attempts and the risk of death. In the regression analyses, univariate analyses were conducted first, and then confounding factors were statistically controlled for. For each variable, the regression coefficient (B), standard error (SE), Wald statistic, odds ratio (OR), 95% confidence intervals, and Nagelkerke R^2^ values were reported.

All statistical tests were considered statistically significant at a p-value level of <0.05.

Because this was a retrospective cohort study, no *a priori* sample size calculation was performed. Instead, all eligible individuals presenting during the predefined study period were included. To assess the adequacy of the final sample, a sensitivity power analysis was conducted. With 454 individuals in the sample group and 451 in the control group (total n = 905), the study had 80% power to detect a small effect size (approximately Cohen’s d = 0.19) at a two-sided alpha level of 0.05.

## 3 Results

**Figure 1:**
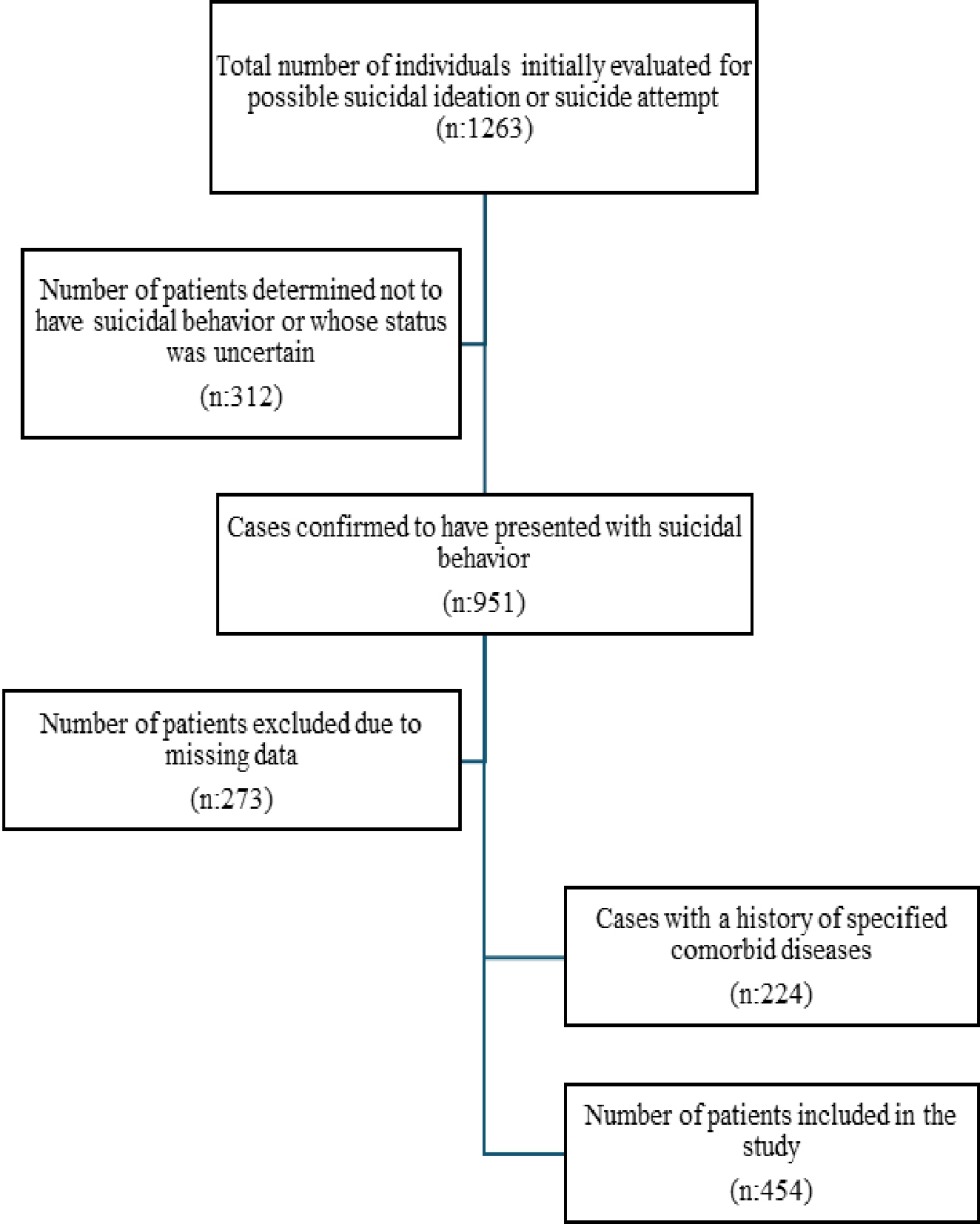
Flowchart illustrating the selection process of participants included in the study.

In our study, as a result of data screening, 454 samples were determined as the control group in accordance with the inclusion and exclusion criteria (**Figure 1**).

**Table 1** compares the demographic characteristics of suicide attempt, suicide ideation, and control groups. The results show that there were no significant differences in age and sex distribution between the groups (p = 0.601 for age and p = 0.291 for sex). In contrast, differences were found between groups in terms of marital status and place of residence. The proportion of married individuals was lower in the suicide attempt and suicide ideation groups (p < 0.001). Similarly, there were differences between groups in terms of rural/urban place of residence (p < 0.001). In the suicide attempt group, the proportion of individuals living in rural areas was higher than in the control group, while in the suicide ideation group, the proportion of individuals living in urban areas was higher (**Table 1**).

**Table 1:** Comparison of demographic characteristics among suicide attempt, suicidal ideation, and control groups.

Median (Q1–Q3)/n (%)	Control (n:451)	Suicidal ideation (n:85)	Suicide attempt (n:369)	Statistics	Effect size (ϵ^2^/φ/V)	P value
Age (years)	31 (29-33)	33 (23-46)	32 (23-42)	H = 1.017	0.001	0.601
Sex				X^2^ = 2.469	0.052	0.291
Female	240 (53.2%)	52 (61.2%)	191 (51.8%)			
Male	211 (46.8%)	33 (38.8%)	178 (48.2%)			
Marital status				X^2^ = 39.389	0.148	<0.001
Married	260 (57.6%)	32 (37.6%)	143 (38.8%)			
Single	174 (38.6%)	50 (50.8%)	192(52%)			
Divorced/Widowed	17 (3.8%)	3 (3.5%)	34 (9.2%)			
Place of residence				X^2^ = 14.982	0.129	<0.001
Rural	223 (49.4%)	36 (42.4%)	224 (60.7%)			
Urban	228 (50.6%)	49 (57.6%)	145 (39.3%)			

H, Kruskal–Wallis test statistic; n, sample size; p, level of statistical significance; Q1, first quartile; Q3, third quartile; χ^2^, chi-square test statistic; ϵ^2^, effect size for Kruskal–Wallis H test; φ, effect size for 2×2 chi-square; V, Cramér’s V for chi-square tests with >2 categories.

**Table 2** compares the various clinical characteristics and outcomes of patients with suicidal ideation and suicide attempts. The distribution of suicide cases according to seasons was found to be similar in both groups, and no statistically significant difference was detected (p = 0.966). However, when the distribution was examined by year, a significant difference was found between the groups (p < 0.001). In particular, the rate of suicide ideation (35.3%) was higher than the rate of suicide attempts (18.2%) in 2022. No significant difference was observed between the groups in terms of psychiatric diagnosis history: In both groups, the majority of patients had at least one known psychiatric diagnosis (p = 0.086). The distribution of specific psychiatric diagnoses was also found to be similar (p = 0.149). Depressive disorder was the most common diagnosis in both groups, and the rates of other diagnostic categories did not differ statistically. The use of psychiatric medications was significantly higher in the suicide attempt group compared to the suicide ideation group (p = 0.039). The presence of comorbid physical illnesses was nearly identical between the two groups (p = 0.995). A history of previous suicide attempts also did not differ between the groups (p = 0.113). Requests for psychiatric consultation were relatively more common among those with suicidal ideation, but this difference did not reach statistical significance (p = 0.061). Differences were found in terms of discharge/treatment outcomes (p < 0.001). Patients who attempted suicide required much more serious medical interventions than those who presented with suicidal ideation. In the attempt group, 23% of cases were admitted to intensive care, 15.2% were treated in the internal medicine ward, and 6% resulted in death. In contrast, admission to the psychiatric ward was more common in the suicide ideation group (21.2%) than in the attempt group (7%), and the rate of transfer to a closed psychiatric ward was also significantly higher in the ideation group (29.4%) than in the attempt group (9.8%). Finally, data on the method of attempt, presented exclusively for the suicide attempt group, indicate that the majority of suicide attempts involved overdose of medication (68.6%). Following this, the most common methods of attempt were the use of firearms/sharp/piercing instruments (19.8%), hanging (6%), and jumping from a height (5.7%) (**Table 2**).

**Table 2:** Comparison of clinical characteristics and outcomes between patients with suicidal ideation and those with suicide attempts.

n (%)	Suicidal ideation (n:85)	Suicidal attempt (n:369)	Statistic (X^2^)	Effect size (φ/V)	P value
Season			0.269	0.024	0.966
Winter	16 (18.8%)	76 (20.6%)			
Spring	28 (32.9%)	112 (30.4%)			
Summer	22 (25.9%)	98 (26.6%)			
Autumn	19 (22.4%)	83 (22.5%)			
Year			18.903	0.204	<0.001
2020	0 (0%)	23 (6.2%)			
2021	14 (16.5%)	53 (14.4%)			
2022	30 (35.3%)	67 (18.2%)			
2023	24 (28.2%)	107 (29%)			
2024	17 (20%)	119 (32.2%)			
History of psychiatric diagnosis			2.947	0.081	0.086
No	34 (40%)	112 (30.4%)			
Yes	51 (60%)	257 (69.6%)			
Psychiatric diagnosis			10.533	0.152	0.149
None	34 (40%)	112 (30.4%)			
Depressive disorder	17 (20%)	85 (23%)			
Anxiety disorder	3 (3.5%)	27 (7.3%)			
Substance use disorder	14 (16.5%)	65 (17.6%)			
Bipolar disorder	10 (11.8%)	19 (5.1%)			
Psychotic disorder	1 (1.2%)	14 (3.8%)			
Personality disorder	3 (3.5%)	25 (6.8%)			
Multiple diagnoses	3 (3.5%)	22 (6%)			
Psychotropic medication use			4.254	0.097	0.039
No	62 (72.9%)	225 (61%)			
Yes	23 (27.1%)	144 (39%)			
Comorbid physical illness			< 0.001	<0.001	0.995
No	74 (87.1%)	324 (87.8%)			
Yes	11 (12.9%)	45 (12.2%)			
History of suicide attempt			2.517	0.075	0.113
No	64 (75.3%)	245 (66.4%)			
Yes	21 (24.7%)	124 (33.6%)			
Psychiatric consultation			3.516	0.088	0.061
No	25 (29.4%)	149 (40.4%)			
Yes	60 (70.6%)	220 (59.5%)			
Outcome/Disposition			73.022	0.401	<0.001
Intensive care unit	2 (2.4%)	85 (23%)			
Internal medicine ward	2 (2.4%)	56 (15.2%)			
General psychiatry ward	18 (21.2%)	26 (7%)			
Closed psychiatric ward	25 (29.4%)	36 (9.8%)			
Refused treatment	21 (24.7%)	73 (19.8%)			
Discharged in good condition	17 (20%)	71 (19.2%)			
Exitus	0 (0%)	22 (6%)			
Type of suicide attempt					
Intoxication		253 (68.6%)			
Hanging		22 (6%)			
Jumping from a height		21 (5.7%)			
Weapons such as firearms or knives		73 (19.8%)			

n, sample size; p, level of statistical significance; χ^2^, chi-square test statistic; φ, effect size for 2×2 chi-square test; V, effect size for chi-square tests with more than two categories (Cramér’s V).

**Table 3** shows that individuals with suicidal ideation and attempts have higher levels of inflammatory markers than the control group, both in the pre-suicide period and at the time of referral. When looking at pre-suicide (PS) values, the suicidal ideation and attempt groups have significantly higher median or mean values than the control group in a number of inflammatory parameters. In particular, the neutrophil count (PS) was significantly increased in patient groups (p < 0.001, SA=SI>HC). No statistically significant difference was found between groups in terms of lymphocyte (PS) levels (p = 0.024). Monocyte (PS) levels also did not differ between groups (p = 0.852). When platelet (PS) counts were examined, a statistically significant difference was found between groups (p = 0.007, SA≥SI≥HC). The neutrophil/lymphocyte ratio (NLR, PS) was significantly increased in patient groups and higher than in the control group (p < 0.001, SA=SI>HC). The platelet/lymphocyte ratio (PLR, PS) also differed between groups (p < 0.001, SA≥SI≥HC). A statistically significant difference was also observed for the monocyte/lymphocyte ratio (MLR, PS) (p = 0.004, SI≥SA≥HC). Additionally, the values of the composite inflammation indices, the Systemic Immune-Inflammation Index (SII, PS) and the Systemic Inflammation Response Index (SIRI, PS), were significantly elevated in the patient groups compared to the control group. (p < 0.001, SA=SI>HC; p < 0.001, SA=SI>HC). A similar pattern was observed when examining inflammatory markers at the time of presentation. The neutrophil count at presentation was again higher in the suicide attempt and suicide ideation groups compared to the control group (p < 0.001, SA=SI>HC). Lymphocyte (at admission) levels did not show significant differences between groups (p = 0.763). Monocyte (at admission) levels showed a significant difference at admission (p = 0.002, SA=SI>HC). Platelet (at admission) values did not show a significant difference between groups (p = 0.171). NLR (at admission) was significantly higher in the suicide ideation and attempt groups compared to the control group (p < 0.001, SA=SI>HC). In contrast, PLR (at admission) distribution did not show a significant difference between groups (p = 0.171). A small difference was detected in MLR (at admission) (p = 0.042, SI≥SA≥ HC). SII (at admission) was significantly higher in the suicide ideation and attempts groups compared to the control group (p < 0.001, SA=SI>HC). SIRI (at admission) values were also elevated in the patient groups (p < 0.001, SA=SI>HC) (**Table 3**).

**Table 3:** Comparison of inflammatory markers among the suicide attempt, suicidal ideation, and control groups.

Mean ± SD/median (Q1–Q3)	Control (n:451)	Suicidal ideation (n:85)	Suicide attempt (n:369)	Statistic	Effect size (η^2^/ϵ^2^)	P value	*Post-hoc*
Neutrophil (PS)	4.08 ± 0.91	5.02 ± 1.21	5.17 ± 1.26	F = 107.570	0.193	<0.001	SA=SI>HC
Lymphocyte(PS)	2.29 ± 0.58	2.13 ± 0.65	2.19 ± 0.69	F = 3.749	0.008	0.024	SA=SI=HC
Monocyte (PS)	0.48 (0.39-0.55)	0.48 (0.39–0.58)	0.47 (0.38–0.58)	H = 0.319	0.002	0.852	
Platelet (PS)	247.93 ± 50.56	255.21 ± 65.08	261.02 ± 66.82	F = 4.995	0.011	0.007	SA≥SI≥HC
NLR (PS)	1.82 (1.52-2.12)	2.28 (1.97–2.78)	2.39 (1.92–2.97)	H = 169.616	0.186	<0.001	SA=SI>HC
PLR (PS)	109.02 (88.63-134.44)	117.22 (95.39–151.41)	120.47 (97.21–155.08)	H = 19.432	0.019	<0.001	SA≥SI≥HC
MLR (PS)	0.21 (0.17-0.25)	0.23 (0.17–0.33)	0.22 (0.18–0.28)	H = 11.122	0.010	0.004	SA≥SI≥HC
SII (PS)	440.46 (350.17-555.75)	577 (457.62–773.95)	603.94 (468.32–785.12)	H =146.638	0.160	<0.001	SA=SI>HC
SIRI (PS)	0.85 (0.66-1.06)	1.16 (0.81–1.56)	1.14 (0.86–1.52)	H = 111.080	0.121	<0.001	SA=SI>HC
Neutrophil	4.05 (3.48–4.69)	5.93 (5.07–6.78)	5.66 (4.32–7.77)	H = 226.902	0.249	<0.001	SA=SI>HC
Lymphocyte	2.24 (1.91–2.66)	2.13 (1.67–2.74)	2.23 (1.68–2.91)	H = 0.541	0.002	0.763	
Monocyte	0.48 (0.39–0.55)	0.52 (0.42–0.61)	0.49 (0.38–0.66)	H = 12.699	0.012	0.002	SA=SI>HC
Platelet	245 (213.5–277.5)	258 (216–302)	250 (205–305)	H = 6.341	0.005	0.042	SA=SI=HC
NLR	1.82 (1.52–2.12)	2.55 (2.03–3.17)	2.37 (1.68–3.77)	H = 131.693	0.144	<0.001	SA=SI>HC
PLR	109.02 (88.63–134.44)	116.67 (96.15–152.46)	112.61 (84.48–142.69)	H = 3.527	0.002	0.171	
MLR	0.21 (0.17–0.25)	0.24 (0.18–0.31)	0.21 (0.16–0.30)	H = 6.339	0.005	0.042	SI≥SA≥HC
SII	440.46 (350.17–555.75)	701.17 (490.25–915.32)	607.33 (404.70–958.69)	H = 122.177	0.133	<0.001	SA=SI>HC
SIRI	0.85 (0.66–1.06)	1.32 (0.99–1.92)	1.23 (0.78–2.00)	H = 120.752	0.132	<0.001	SA=SI>HC

F, One-way ANOVA test statistic; H, Kruskal–Wallis H test statistic; n, sample size; p, statistical significance level; Q1, first quartile; Q3, third quartile; SD, standard deviation; ϵ^2^, effect size for Kruskal–Wallis H test; η^2^, effect size for ANOVA.

NLR: Neutrophil-to-Lymphocyte Ratio, PLR: Platelet-to-Lymphocyte Ratio, MLR: Monocyte-to-Lymphocyte Ratio, SII: Systemic Immune-Inflammation Index, SIRI: Systemic Inflammation Response Index, PS: Pre-suicide, SA: Suicidal Attempt, SI: Suicidal ideation, HC: Health control.

**Table 4** presents a comparison of inflammatory marker values in patients who attempted suicide and those with suicidal ideation, both within the same group and between pre-admission and admission. Significant changes were observed in most inflammatory parameters in the suicide attempt group. The neutrophil count increased significantly during the presentation compared to before the attempt (p < 0.001). Similarly, a statistically significant increase was observed in the lymphocyte count (p < 0.001). Monocyte levels significantly increased after the suicide attempt (*p* = 0.002). In contrast, platelet counts did not show a statistically significant change between the pre- and post-attempt periods (*p* = 0.534). Regarding inflammatory indices, NLR significantly decreased following the attempt (*p* = 0.003), and PLR also showed a marked reduction (*p* < 0.001). MLR remained virtually unchanged between the two time points (*p* = 0.980). SII was found to be significantly elevated after the attempt (*p* = 0.015), and similarly, SIRI showed a pronounced increase following the suicide attempt (*p* < 0.001).

**Table 4:** Changes in inflammatory markers before and at the time of admission in patients with suicide attempts and suicidal ideation.

Mean ± SD/ median (Q1–Q3)	Suicide attempt (PS) (n:369)	Suicide attempt (at admission) (n:369)	Statistic	Effect size (*d*/r)	P value	Suicidal ideation (PS) (n:85)	Suicidal ideation (at admission) (n:85)	Statistic	Effect size (*d*/r)	P value
Neutrophil	5.17 ± 1.26	5.66 (4.32–7.77)	t = -7.127	0.371	<0.001	4.93 (4.20-5.96)	5.93 (5.05-6.84)	Z = -4.868	0.528	<0.001
Lymphocyte	2.19 ± 0.69	2.23 (1.68–2.91)	t =-4.400	0.229	<0.001	2.13 ± 0.65	2.23 (1.68–2.91)	t = -1.839		0.069
Monocyte	0.47 (0.38–0.58)	0.49 (0.38–0.66)	Z=-3.103	0.161	0.002	0.48 (0.39–0.58)	0.52 (0.42–0.61)	Z = -2.837	0.308	0.005
Platelet	261.02 ± 66.82	259-76.45	t = 0.623	0.032	0.534	249 (209-300)	258 (214.5-304.5)	Z = -1.684	0.183	0.092
NLR	2.39 (1.92–2.97)	2.37 (1.68–3.77)	Z=-3.018	0.157	0.003	2.28 (1.97–2.78)	2.55 (2.03–3.17))	Z = -2.456	0.266	0.014
PLR	120.47 (97.21–155.08)	112.61 (84.48–142.69)	Z=-3.564	0.185	<0.001	117.22 (95.39–151.41)	116.67 (96.15–152.46)	Z = -0.843	0.091	0.399
MLR	0.22 (0.18–0.28)	0.21 (0.16–0.30)	Z=-0.026	0.001	0.980	0.23 (0.17–0.33)	0.24 (0.18–0.31)	Z = -1.571	0.170	0.116
SII	603.94 (468.32–785.12)	607.33 (404.70–958.69)	Z=-2.426	0.126	0.015	577 (457.62–773.95)	701.17 (490.25–915.32)	Z = -3.135	0.340	0.002
SIRI	1.14 (0.86–1.52)	1.23 (0.78–2.00)	Z=-4.155	0.216	<0.001	1.16 (0.81–1.56)	1.32 (0.99–1.92)	Z = -3.267	0.354	0.001

n, sample size; p, level of statistical significance; Q1, first quartile; Q3, third quartile; SD, standard deviation; t, paired samples *t*-test statistic; Z, Wilcoxon test statistic; d, effect size for paired samples *t*-test; r, effect size for Wilcoxon test.

NLR: Neutrophil-to-Lymphocyte Ratio, PLR: Platelet-to-Lymphocyte Ratio, MLR: Monocyte-to-Lymphocyte Ratio, SII: Systemic Immune-Inflammation Index, SIRI: Systemic Inflammation Response Index, PS: Pre-suicide.

The neutrophil count was significantly elevated at the time of admission in the suicidal ideation group (*p* < 0.001). The lymphocyte count did not show a statistically significant change between the pre- and post-ideation periods (*p* = 0.069). A significant increase in monocyte levels was also detected at admission (*p* = 0.005), while platelet counts remained unchanged (*p* = 0.092). NLR was another parameter that significantly increased following suicidal ideation (*p* = 0.014). In contrast, PLR values remained stable (*p* = 0.399), and no significant change was observed in MLR (*p* = 0.116). Both SII (*p* = 0.002) and SIRI (*p* = 0.001) showed significant increased at the time of admission (**Table 4**).

**Table 5** presents the role of inflammatory markers prior to the application in predicting suicide attempts, evaluated using logistic regression analysis. A binary logistic regression model was used, with the dependent variable being the occurrence of a suicide attempt and the independent variables being the complete blood inflammatory parameters from the period prior to the suicide attempt. In the analysis predicting the risk of suicide attempts based on inflammatory markers prior to the attempt, all parameters except monocyte count were found to have a statistically significant effect. In particular, an increase in neutrophil count (univariate OR = 2.617, p < 0.001) significantly elevates the risk of suicide attempt, while high lymphocyte levels are associated with a reduced risk (univariate OR = 0.777, p = 0.025). Among inflammation indices, NLR (univariate OR = 4.554), PLR (univariate OR = 1.009), MLR (univariate OR = 25.223), SII (univariate OR = 1.005), and SIRI (univariate OR = 6.586) also significantly increase the risk (all p < 0.001). When confounding factors (age, gender, marital status, place of residence, season of application, year of application, psychiatric diagnoses, use of psychiatric medications, and known comorbidities) were included in the model, statistical significance was maintained for variables other than PLT and MLR (**Table 5**). **Table 6** examines the prediction of suicide attempt risk based on inflammatory markers at the time of application. A binary logistic regression model was used, with suicide attempt status as the dependent variable and complete blood inflammatory parameters at the time of application as independent variables. In the logistic regression analysis conducted using inflammatory markers at the time of presentation, all parameters examined significantly predicted the risk of suicide attempt. Elevated neutrophil (univariate OR = 2.217, p < 0.001) and monocyte (univariate OR = 7.721, p < 0.001) counts were associated with a significant increase in risk. Elevated inflammatory markers, particularly NLR (univariate OR = 2.608) and especially MLR (univariate OR = 19.900), significantly increased the likelihood of suicide attempts (p < 0.001). Additionally, elevated SIRI (univariate OR = 4.464, p < 0.001) and SII (univariate OR = 1.003, p < 0.001) levels were also associated with a statistically significant increase in risk. Platelet (univariate OR = 1.003, p = 0.014) and lymphocyte (univariate OR = 1.225, p = 0.012) counts were also found to be statistically significant, albeit with smaller effect sizes. When confounding factors (age, gender, marital status, place of residence, season of presentation, year of presentation, psychiatric diagnoses, use of psychiatric medications, and known comorbidities) were included in the model, the values for neutrophils, NLR, SII, and SIRI retained their statistical significance, whereas the other hematological parameters were no longer statistically significant (**Table 6**).

**Table 5:** Pre-attempt inflammatory markers predicting suicide attempt: binary logistic regression analysis.

Variable	B	SE	Wald	p	Univariate OR	Adjusted OR*	95% CI	R^2^
Neutrophil	1.068	0.163	42.914	<0.001	2.617	2.909	2.114-4.005	0.854
Lymphocyte	-0.680	0.257	7.019	0.008	0.777	0.507	0.307-0.838	0.827
Monocyte	-2.219	1.285	2.981	0.084	2.112	0.109	0.009-1.350	0.824
Platelet	0.004	0.002	2.494	0.114	1.004	1.004	0.999-1.009	0.824
NLR	1.865	0.273	46.535	<0.001	4.554	6.456	3.778-11.034	0.863
PLR	0.012	0.004	11.605	<0.001	1.009	1.012	1.005-1.019	0.830
MLR	2.270	1.893	1.437	0.231	25.223	9.680	0.237-395.843	0.823
SII	0.006	0.001	44.068	<0.001	1.005	1.006	1.004-1.008	0.860
SIRI	1.954	0.365	28.735	<0.001	6.586	7.058	3.455-14.421	0.842

B, Unstandardized regression coefficient; SE, Standard error; Wald, Wald chi-square test statistic; OR, Multivariate odds ratio; CI, Confidence interval; R^2^, Nagelkerke R-squared; p, Level of statistical significance. NLR, Neutrophil-to-Lymphocyte Ratio; PLR, Platelet-to-Lymphocyte Ratio; MLR, Monocyte-to-Lymphocyte Ratio; SII, Systemic Immune-Inflammation Index; SIRI, Systemic Inflammation Response Index.

*Confounding factors: Age, gender, marital status, place of residence, season of presentation, year of presentation, psychiatric diagnoses, use of psychiatric medications, known comorbid conditions.

**Table 6:** Inflammatory markers at admission predicting suicide attempt: binary logistic regression analysis.

Variable	B	SE	Wald	p	Univariate OR	Adjusted OR*	95% CI	R^2^
Neutrophil	1.341	0.177	57.157	<0.001	2.217	3.825	2.701-5.415	0.888
Lymphocyte	0.142	0.206	0.475	0.491	1.225	1.152	0.770-1.725	0.823
Monocyte	1.435	1.081	1.762	0.184	7.721	4.198	0.505-34.912	0.823
Platelet	0.003	0.003	1.089	0.297	1.003	1.003	0.998-1.008	0.823
NLR	1.546	0.227	46.297	<0.001	2.608	4.691	3.006-7.323	0.869
PLR	0.005	0.003	1.915	0.166	1.004	1.005	0.998-1.011	0.824
MLR	2.703	1.475	3.358	0.067	19.900	14.922	0.829-268.743	0.825
SII	0.005	0.001	38.279	<0.001	1.003	1.005	1.003-1.006	0.860
SIRI	1.971	0.355	30.790	<0.001	4.464	7.175	3.577-14.391	0.856

B, Unstandardized regression coefficient; SE, Standard error; Wald, Wald chi-square test statistic; OR, Multivariate odds ratio; CI, Confidence interval; R^2^, Nagelkerke R-squared; p, Level of statistical significance. NLR, Neutrophil-to-Lymphocyte Ratio; PLR, Platelet-to-Lymphocyte Ratio; MLR, Monocyte-to-Lymphocyte Ratio; SII, Systemic Immune-Inflammation Index; SIRI, Systemic Inflammation Response Index.

*Confounding factors: Age, gender, marital status, place of residence, season of presentation, year of presentation, psychiatric diagnoses, use of psychiatric medications, known comorbid conditions.

**Table 7** presents the results of a logistic regression analysis examining the variables predicting the risk of death in the group of patients who attempted suicide. Here, only suicide attempt cases were considered, and the dependent variable was whether death occurred after the attempt. The results of the logistic regression analysis predicting the risk of death in suicide attempt cases revealed significant effects of certain demographic/clinical factors and inflammatory parameters. Male gender (univariate OR = 25.414, p = 0.002), being single (univariate OR = 4.700, p = 0.045), and being divorced/widowed (univariate OR = 21.692, p < 0.001) were significantly associated with a higher mortality risk. Having received psychiatric consultation demonstrated a protective effect that significantly reduced the risk of death (univariate OR = 0.059, p < 0.001). The risk of death was approximately three times higher in cases with known comorbid conditions (univariate OR = 2.962, p = 0.033). Inflammatory markers from the pre-suicide period, including elevated monocytes (univariate OR = 13.708, p = 0.030) and lymphocytes (univariate OR = 1.785, p = 0.044), also significantly predict the risk of death. Similarly, elevated levels of neutrophils (univariate OR = 1.156, p < 0.001), lymphocytes (univariate OR = 1.459, p = 0.006), and monocytes (univariate OR = 13.113, p < 0.001) counts at presentation, along with elevated NLR (univariate OR = 1.166, p = 0.002), MLR (univariate OR = 12.202, p = 0.007), and SIRI (univariate OR = 1.229, p = 0.001) values significantly increased the risk of mortality. On the other hand, no statistically significant effect of platelet count or PLR levels on mortality was detected (p > 0.05). After adjusting for confounding factors (age, gender, marital status, place of residence, season of presentation, year of presentation, psychiatric diagnoses, use of psychiatric medications, known comorbid conditions, type of suicide attempt, and history of suicide), the pre-attempt PLR became significant, while pre-attempt monocytes, post-attempt monocytes, NLR, MLR, SII, and SIRI values lost their significance; however, pre-attempt lymphocytes and post-attempt neutrophil and lymphocyte values retained their significance (**Table 7**).

**Table 7:** Binary logistic regression analysis of predictors of mortality in patients with suicide attempts.

Variable	Reference	B	SE	Wald	p	Univariate OR	Adjusted OR*	95% CI	R^2^
Sex (Male)	Female	3.235	1.029	9.882	0.002	25.414		3.381-191.042	
Marital status	Married								
Single		1.548	0.772	4.018	0.045	4.700		1.035-21.341	
Divorced/widowed		3.077	0.819	14.119	<0.001	21.692		4.358-107.977	
Psychiatric consultation (Yes)	No	-2.827	0.750	14.215	<0.001	0.059		0.014-0.257	
Known comorbidity (Yes)	No	1.086	0.508	4.568	0.033	2.962		1.094-8.015	
Neutrophil (PS)		0.315	0.278	1.283	0.257	1.168	1.371	0.794-2.366	0.514
Lymphocyte (PS)		1.009	0.456	4.908	0.027	1.785	2.744	1.123-6.702	0.540
Monocyte (PS)		0.391	1.845	0.045	0.832	13.708	1.478	0.040-54.935	0.507
Platelet (PS)		-0.003	0.006	0.373	0.541	0.996	0.997	0.986-1.008	0.509
NLR (PS)		-0.387	0.363	1.135	0.287	0.856	0.679	0.333-1.384	0.514
PLR (PS)		-0.019	0.009	4.254	0.039	0.989	0.981	0.964-0.999	0.537
MLR (PS)		-4.992	3.823	1.705	0.192	1.359	0.007	0.000-12.205	0.518
SII (PS)		-0.002	0.002	1.993	0.158	0.999	0.998	0.995-1.001	0.519
SIRI (PS)		-0.500	0.592	0.713	0.399	1.052	0.607	0.190-1.935	0.511
Neutrophil		0.141	0.062	5.150	0.023	1.156	1.151	1.019-1.300	0.542
Lymphocyte		0.489	0.202	5.886	0.015	1.459	1.631	1.099-2.421	0.544
Monocyte		1.983	1.206	2.705	0.100	13.113	7.262	0.684-77.131	0.525
Platelet		0.002	0.004	0.184	0.668	1.000	1.002	0.993-1.010	0.507
NLR		0.153	0.106	2.064	0.151	1.166	1.165	0.946-1.435	0.523
PLR		-0.008	0.007	1.663	0.197	0.995	0.992	0.979-1.004	0.517
MLR		1.742	1.984	0.771	0.380	12.202	5.709	0.117-278.591	0.511
SII		<0.001	<0.001	1.347	0.246	1.000	1.000	1.000-1.001	0.519
SIRI		0.225	0.124	3.306	0.069	1.229	1.252	0.983-1.595	0.533

B, Unstandardized regression coefficient; SE, Standard error; Wald, Wald chi-square test statistic; OR, Multivariate odds ratio; CI, Confidence interval; R^2^, Nagelkerke R-squared; p, Level of statistical significance. NLR, Neutrophil-to-Lymphocyte Ratio; PLR, Platelet-to-Lymphocyte Ratio; MLR, Monocyte-to-Lymphocyte Ratio; SII, Systemic Immune-Inflammation Index; SIRI, Systemic Inflammation Response Index; PS, Pre-suicide.

*Confounding factors: Age, gender, marital status, place of residence, season of presentation, year of presentation, psychiatric diagnoses, use of psychiatric medications, known comorbid conditions, type of suicide attempt, history of suicide.

## 4 Discussion

In our study, inflammatory markers were calculated from CBC values obtained within one year prior to and at the time of presentation in individuals who presented to the emergency department with suicide attempts and ideation. This study is one of the few studies examining the relationship between suicidal behavior and inflammatory markers in a two-time point analysis. A key strength of this study is the evaluation of inflammatory markers at two distinct time points, allowing a more comprehensive assessment of both baseline and acute inflammatory processes associated with suicidal behavior. According to the findings of our study, inflammation parameters are elevated in the year preceding suicidal behavior and continue to be elevated at the time of referral. We also found that male gender, additional physical illness, and elevated inflammation parameters increase the risk of suicide-related death. Our findings suggest a potential association between suicidal behavior and chronic inflammation. However, these findings should be interpreted with caution due to the retrospective design and the potential influence of confounding variables.

In our study, the rate of unmarried individuals with suicidal behavior was found to be significantly higher than that of the control group. In the literature, this finding may be explained by feelings of loneliness, lack of social support, and emotional belonging (9, 32). In addition, the present study showed that suicide attempts were more common among people living in rural areas than among those living in cities. The literature suggests that this situation may be related to limited access to health services, social isolation, and increased stress factors (10).

When comparing suicide attempts and suicidal ideation, we found some differences in discharge and treatment outcomes. The suicide attempt group had a higher need for intensive care, treatment in internal medicine departments, and mortality rates. This likely reflects the severity of medical complications resulting from the suicide attempt. The suicide ideation group, on the other hand, was treated mainly by admission to open and closed psychiatric wards. This may be because patients presenting with suicidal ideation require more psychiatric monitoring and psychosocial intervention (33). Our study found a higher use of psychiatric medications in the suicide attempt group. This may reflect these individuals having previously been diagnosed, being resistant to treatment, or having a clinically severe patient profile. It is known in the literature that treatment-resistant mental disorders significantly increase the risk of suicide (34, 35). These findings are in line with previous work from our group, which demonstrated that patients admitted to intensive care following suicide attempts tend to exhibit more severe clinical presentations and higher mortality risk, supporting the role of systemic factors in determining outcomes (30).

In the present study, the rate of completed suicide was substantially higher in men. The literature reports this rate to be between 2 and 4 times higher (36, 37). An extensive sample study found that the mortality rate of suicide attempts was 3.3% for women and 14.7% for men (38). This may be explained by men’s low help-seeking behavior, their preference for more lethal methods, and their delayed access to the healthcare system (37, 38).

In line with the literature, our study revealed a link between unmarried status and a higher risk of death following a suicide attempt (9). Our study revealed a significantly higher post-suicide mortality rate among individuals who did not receive psychiatric consultation. These findings may reflect the priority given to medical stabilization before psychiatric intervention in life-threatening suicide attempts. On the other hand, in suicide attempts where psychiatric intervention was provided, psychological stabilization may have been achieved, treatment compliance may have improved, and agitation may have decreased. This situation may have contributed to reduced mortality (39). In addition, our study found that the presence of physical illness increased the risk of suicide-related death by approximately three-fold. A review of the literature indicates that the presence of physical illnesses that significantly limit daily activities increases the risk of suicide-related death by approximately three-fold (40).

In our study, individuals exhibiting suicidal behavior had significantly higher levels of neutrophil, platelet, NLR, MLR, PLR, SII, and SIRI in CBCs taken for various clinical reasons in the year prior to presenting to the emergency department compared to the control group. These findings suggest that suicidal behavior may be associated with both acute and chronic inflammatory processes. Comprehensive reviews have reported that chronic inflammation may increase suicidal tendencies by causing microglial activation in the brain, increasing specific cytokines, and neurotransmitter imbalances (41–43). The literature primarily establishes the relationship between suicidal behavior and inflammation during or after the attempt. Our study suggests that inflammation parameters may serve as potential early biological indicators weeks or even months before suicidal behavior. Additionally, it is possible that inflammatory markers may increase in response to chronic stressors. Wu et al. (2025) reported that high SII scores may be significantly associated with subsequent suicide attempts in patients with major depressive disorder (27). However, the number of such studies is quite limited. Nevertheless, they suggest that inflammatory markers may also be used prospectively for predictive purposes.

In individuals presenting to the emergency department with suicidal behavior, inflammatory parameters were found to be elevated at the time of presentation, similar to previous periods. In particular, elevated levels of neutrophils, monocytes, NLR, MLR, SII, and SIRI, in conjunction with an acute stress response, may reflect an underlying biological vulnerability. A study conducted in 2024 reported that patients presenting to the emergency department with suicide attempts had significantly higher levels of the same inflammatory parameters compared to the control group (14). These findings are associated with the acute stress response in suicide attempts, mobilizing proinflammatory cells such as neutrophils and monocytes (14, 15). However, in our study, the fact that these parameters were also found to be high prior to suicide suggests that this condition is not merely an acute stress response but rather indicates the presence of an underlying chronic inflammatory process.

Many studies have compared individuals who have attempted suicide to those who have not. Notably, several retrospective cohort studies suggest that high values of the NLR and the MLR may predict future suicide attempts (44, 45). Recent studies have reported that composite inflammation indices such as SII and SIRI may be significantly associated with suicide risk (27, 28, 46). These indices are calculated from cell values such as neutrophils, monocytes, lymphocytes, and platelets and can reflect the inflammatory load holistically. In our study, the high levels of these indices both before suicidal behavior and at the time of referral support their potential clinical applicability in suicide risk assessment. Among individuals in the suicide attempt group, neutrophil, lymphocyte, monocyte counts as well as SII and SIRI values were significantly increased at the time of emergency admission compared to the preceding period, while NLR and PLR levels were significantly decreased.

This paradoxical pattern is thought to reflect the complex and multidimensional nature of the immune response. The simultaneous elevation of both neutrophil and lymphocyte counts may explain the observed reductions in NLR and PLR. Neutrophils are cells that are rapidly activated by the acute stress response resulting from suicidal behavior, while lymphocytes are cells of the adaptive immune system (47, 48). The fact that monocytes were significantly elevated at the time of application in both the suicide ideation and suicide attempt groups indicates that low-grade systemic inflammation is also active. In our study, SII was found to be elevated at the time of application in both groups. Research has shown that systemic immune activation is common and significant in suicidal behavior (13, 49). This increase indicates that SII is also an indicator of acute biological stress. SIRI increased significantly in both groups, although the increase was higher in the intervention group. The increase in this index in both groups indicates that immunity is activated both cellularly and functionally.

In our study, all parameters measured prior to admission, including neutrophil, lymphocyte, NLR, MLR, PLR, SII, and SIRI, were found to be significantly associated with suicide attempt risk. In particular, NLR and SIRI show a significant association with suicide attempts. SIRI may reflect the biological burden of suicidal behavior due to its association with both neutrophil and monocyte increases and lymphocyte suppression. There are only a limited number of studies on SIRI and suicide risk rates. In our study, higher neutrophil counts were associated with an increased likelihood of suicide (OR = 2.6), while higher lymphocyte levels were associated with a reduced likelihood (approximately 22%). Large-scale studies have shown that an increase in NLR increases the likelihood of suicide attempts by 1.6 to 3.5 times (45, 50). This suggests that these parameters can be integrated into psychiatric assessment and used as helpful and supportive criteria in risk classification. Importantly, after adjustment for confounding variables, the statistical significance of several inflammatory parameters was attenuated, suggesting that these markers may not act as independent predictors but rather reflect broader clinical and biological processes. One of the most striking findings in our study is that MLR scores prior to suicide were associated with a markedly increased risk. In addition, MLR scores were significantly higher in the suicide attempt group than in the contemplation group. The results may reflect a psychopathological spectrum that increases from normal to attempt. Previous studies have noted that there is insufficient evidence regarding the relationship between MLR and suicide risk and that further research is needed in this area (18). Our findings suggest that the suicide-inflammation relationship may also be associated with deeper and chronic immune responses originating from monocytes. This finding should be interpreted cautiously, as the effect size decreased after multivariable adjustment, suggesting that the observed association may be confounded.

In this study, logistic regression analysis examining the association between inflammatory parameters measured at the time of admission and suicide attempts revealed that elevated MLR levels were associated with a substantially increased risk, with approximately a 20-fold increase in the odds of attempting suicide, while elevated monocyte counts were associated with an 8-fold increase. These variables were identified as the most significant independent predictors of suicide attempts. Monocytes are known to play a central role in the peripheral inflammatory response and, by crossing the blood-brain barrier, can exert pro-inflammatory effects within the central nervous system, potentially contributing to neuroinflammatory mechanisms underlying suicidal behavior (51). In animal models, the migration of monocytes to the brain and their proinflammatory response have been shown to contribute to the development of anxiety and stress-related psychiatric disorders (51, 52).

Some inflammatory values were found to be significantly elevated in cases resulting in suicide related death before suicide and at the time of referral. In particular, elevated monocyte and lymphocyte counts prior to suicide may indicate that these individuals were under inflammatory stress before suicide. High inflammatory parameters at the time of application indicate high systemic immune activation. Studies have shown that markers such as NLR, MLR, and SII are particularly high in completed suicides (15, 27, 53). In addition, elevated IL-6, CRP, and NLR levels have been repeatedly associated with suicide lethality (5, 12, 54). Although studies have shown that elevated SIRI levels increase the risk of death from many diseases, there are only a limited number of studies investigating the risk of death by suicide (23). In our study, we found that elevated monocyte and MLR levels increased the risk of suicide-related death by 12–13 times. The literature reports that elevated MLR and monocyte levels are independent risk factors for mortality in the general population and chronic diseases (55, 56). However, studies on its association with increased risk of suicide are quite limited. Similarly, the attenuation of associations in mortality analyses after adjustment suggests that inflammatory markers alone may not fully account for suicide-related outcomes and that clinical variables likely play a significant role.

### 4.1 Limitations

The present study has several limitations. First, due to its retrospective cohort design, causal relationships cannot be established. Second, the data were collected from three hospitals within a single province, which may limit the generalizability of the findings. Third, the use of electronic medical records may have resulted in incomplete data regarding psychiatric diagnoses, suicide severity, and treatment-related variables. In addition, potential confounding factors such as substance use, comorbid medical conditions, and medication use could not be fully controlled. The lack of standardized timing for blood sample collection also limits the ability to distinguish between acute and chronic inflammatory responses. Finally, the absence of long-term follow-up data restricts the evaluation of the prognostic value of inflammatory markers.

## 5 Conclusion

The present study provides evidence suggesting that suicidal behavior may be linked to a biological substrate, particularly chronic low-grade inflammation. In the present study, specific hematological inflammatory markers were found to be significantly elevated both prior to suicidal behavior and at the time of presentation compared to controls. The fact that these markers were elevated weeks or even months before clinical symptoms emerged suggests that they could serve as early warning indicators for suicide risk assessment.

NLR, MLR, and SIRI values at the time of referral showed the strongest predictive value for suicide risk. Elevated SIRI and SII values may indicate acute stress response in addition to chronic inflammation. These findings suggest that they may serve as practical, accessible markers for identifying high-risk individuals. These markers may be integrated into clinical risk assessment; however, further prospective studies are required to confirm their predictive value.

From a biological perspective, these findings may be explained by the role of systemic inflammation in modulating neurobiological pathways associated with suicidal behavior. Chronic inflammation may contribute to microglial activation, cytokine imbalance, and neurotransmitter dysregulation. Therefore, inflammatory markers may reflect underlying neuroimmune mechanisms rather than merely acute stress responses.

## Data Availability

The datasets generated and/or analysed during the current study are not publicly available due to ethical restrictions and patient confidentiality, but are available from the corresponding author on reasonable request.
